# Lymphocytes as an Indicator for Initial Kidney Function: A Single Center Analysis of Outcome after Alemtuzumab or Basiliximab Induction

**DOI:** 10.1155/2015/985460

**Published:** 2015-06-11

**Authors:** Annemarie Weissenbacher, Theresa Hautz, Michael Kimelman, Rupert Oberhuber, Hanno Ulmer, Claudia Bösmüller, Manuel Maglione, Stefan Schneeberger

**Affiliations:** ^1^Department of Visceral, Transplant and Thoracic Surgery, Center of Operative Medicine, Innsbruck Medical University, 6020 Innsbruck, Austria; ^2^Department of Medical Statistics, Informatics and Health Economics, Innsbruck Medical University, 6020 Innsbruck, Austria

## Abstract

Alemtuzumab, an anti-CD52 T-cell and B-cell depleting monoclonal antibody, is established for induction therapy in renal transplantation (KTx). We herein provide a comparative analysis between alemtuzumab and basiliximab induction therapy and correlate lymphocyte depletion and recovery with the clinical course after KTx. This is a single center retrospective analysis of 225 patients/consecutive kidney transplantations treated with alemtuzumab for lymphocyte depletion and 205 recipients treated with basiliximab. Mean lymphocyte counts were 22.8 ± 9.41% before Tx and 2.61 ± 3.11% between week 1 and week 3 in the alemtuzumab group and 23.77 ± 10.42% before Tx and 13.92 ± 8.20% in the basiliximab group. Delayed graft function (DGF), cytomegalovirus (CMV) status, and recipient age showed a significant correlation with lymphocyte counts in the alemtuzumab group only. The outcome was read in reference to the velocity of lymphocyte recovery and in comparison to the control group. Lymphocyte counts early after transplantation, following alemtuzumab treatment, could be identified as a predictive factor for kidney function early after transplantation. A detailed analysis of phenotype and function of lymphocytes after alemtuzumab induction together with a correlation with the clinical course is warranted.

## 1. Introduction

The use of induction therapy in kidney transplantation reduces early rejection rates and graft loss in the first year after transplantation. In patients with high-risk immune status such as patients undergoing retransplantation, sensitized patients, and recipients with a high number of donor-specific T-cell precursors, induction therapy is increasingly used [1]. Further, alemtuzumab induction has been employed in trials aiming at minimization of maintenance immunosuppression [2–4]. The most frequently used induction agents in kidney transplantation are rabbit antithymocyte globulin (ATG), a polyclonal depleting antibody, and basiliximab, a nondepleting monoclonal antibody targeting the interleukin-2 receptor. Daclizumab, a second humanized monoclonal antibody of the IL-2 receptor of T-cells, is marketed in the United States but not in Europe. Alemtuzumab is an IgG1 humanized rat monoclonal antibody that binds to CD52, an antigen found on B- and T-lymphocytes, monocytes, macrophages, dendritic cells, and natural killer cells [5]. Alemtuzumab has been used for treatment of hematologic malignancies, bone marrow transplantation, and autoimmune diseases and as an induction agent in solid organ transplantation [6–9]. Alemtuzumab first received broad attention in transplantation, when Calne et al. reported that it may promote “prope tolerance,” a status in which graft acceptance is achieved with only a minimal dose of long-term immunosuppression that may even be withdrawn at one point [10, 11]. Several studies demonstrated that leukocyte depletion with alemtuzumab at the time of transplantation may facilitate reduction of maintenance immunosuppression. The effectiveness and safety profile of alemtuzumab compare well with those of other induction agents [12, 13] with respect to both antibody mediated and cellular rejection [14, 15].

The recovery of lymphocytes after depletion with alemtuzumab has been investigated in a number of trials. B-cells return within 2 to 12 months and T-lymphocytes may remain low for years [16]. Sageshima et al. retrospectively analyzed lymphocyte phenotypes of kidney transplant recipients, who received different antibodies and antibody combinations for induction therapy. Alemtuzumab and the combination with thymoglobulin lead to a greater extent of CD4+ T-cell suppression than thymoglobulin alone or a combination with daclizumab. The effect persists up to 3 years and lymphocytes remain at about 40% of baseline. CD8+ T-cell showed a similar trend but recovered more rapidly to baseline. CD19+ B-cells returned to baseline at 2 months [17, 18].

Herein, we correlate the outcome as well as recipient factors with lymphocyte recovery after induction with alemtuzumab and basiliximab.

## 2. Patients and Methods

### 2.1. Patients and Data Collection

This is a single center retrospective analysis of 430 kidney transplantations performed between January 1, 2004, and December 31, 2011. The T- and B-cell complement-dependent cytotoxic (CDC) crossmatch was negative in all patients. Alemtuzumab (30 mg) was started just prior to graft reperfusion (*n* = 225). Basiliximab (20 mg) was given within 2 hours after start of surgery and repeated on day 4 (*n* = 205). Lymphocyte counts were reviewed and patients assessed retrospectively in reference to the velocity of lymphocyte recovery. Relative lymphocyte counts at different periods of time, which were included in the analysis, were as follows: pre-Tx, day 7 to 3 weeks, 3 weeks to 3 months, and 3 to 6 months after KTx.

Demographic data include cause of renal failure, age, gender, history of a prior transplant, cytomegalovirus (CMV) status, panel reactive antibodies (PRAs), mismatches in HLA-A, HLA-B, and HLA-DR, biopsy proven acute rejection (BPAR), pretransplant serum creatinine, and posttransplant serum creatinine on the 1st, 3rd, 7th, 14th, and 21st day. Delayed graft function (DGF) is defined according to UNOS criteria as the need for at least 1 dialysis within the first week after transplantation except for single dialysis for high potassium or volume overload. The relative lymphocyte count was assessed at 4 different time points in the alemtuzumab group and twice (days 1–6 and 1 to 3 weeks) in the basiliximab group.

Criteria for rejection were positive biopsy and/or rise in serum creatinine >0.5 mg/dL and reduced graft blood flow determined by Doppler ultrasonography.

The study was approved by the Institutional Review Board of the Innsbruck Medical University (UN4632; 310/4.11; February 24, 2012).

### 2.2. Statistical Analysis

Statistical analysis was performed with SPSS 17.0 software (SPSS Inc., Chicago, IL, USA) and GraphPad Prism 4.0 (GraphPad Software, La Jolla, CA, USA). Analysis of variance for repeated testing with measurement time as within-subject factor and with age, CMV status, and DGF was performed. To test for univariate differences in categorical variables, the Pearson chi-square test or Fisher exact test (when appropriate) was applied. Continuous variables were tested with the Student *t*-test or Mann-Whitney *U* test (if assumption of Gaussian distribution was not fulfilled). Thereby, the selection of variables was based on univariate comparisons (entry criteria: *p* < 0.05) and clinical relevance. Graft and patient survival was calculated using Kaplan-Meier estimates. Values if not otherwise indicated are means ± SD.

## 3. Results and Discussion

Recipient characteristics are shown in Table 1.

Most common causes of renal failure were glomerulonephritis (31.55% versus 33.17% in the control group), diabetes mellitus (27.12% versus 28.29% in the control group), and polycystic kidney disease (9.78% versus 11.22% in the control group). There were no significant differences between the two groups. More than 60% of all recipients in both groups were male (62.22% versus 66.34% in the alemtuzumab versus basiliximab group, *p* = 0.495). Recipients receiving alemtuzumab were significantly younger than the patients in the basiliximab group (48.46 ± 12.37 versus 59.57 ± 13.16, *p* < 0.0001). Further, the alemtuzumab group included significantly more retransplantations than the basiliximab group (18.22% versus 8.29%, *p* = 0.0021). In the alemtuzumab group, there were significantly more patients with PRAs (*p* = 0.021) and the mean PRAs at the time of transplantation were higher in this group than in the group of recipients who received basiliximab (22.93 ± 26.99% versus 15.65 ± 4.25%, *p* = 0.189) but without reaching statistical significance, indicating the retrospective nonrandomized nature of this trial. Recipients in the alemtuzumab group had significantly more HLA mismatches in locus B than the patients in the control group, *p* = 0.019.

DGF rate in the alemtuzumab group was significantly lower than in the basiliximab group, 27.56% versus 38.54%, *p* = 0.0154.

For maintenance immunosuppression tacrolimus, ciclosporin, mycophenolate mofetil (MMF), and mycophenolic acid (MPA) were used in both groups: for the alemtuzumab group 73.4% tacrolimus, 26.6% ciclosporin, 91.2% MMF, and 8.8% MPA and in the basiliximab group 72.1% tacrolimus, 27.9% ciclosporin, 89.4% MMF, and 10.6% MPA. There were no significant differences either between the induction treatment groups or in the occurrence of DGF between the calcineurin inhibitors, MMF, and MPA.

### 3.1. Lymphocyte Counts, DGF, and CMV Status

The pre-Tx lymphocyte counts were not significantly different in the two groups, *p* = 0.41. Among all factors analyzed, DGF, age, and CMV status showed a significant correlation with the lymphocyte count at different time points in the alemtuzumab group. Mean lymphocyte counts in this group were 22.8 ± 9.41% before Tx; 2.61 ± 3.11% between day 7 and week 3; 6.98 ± 6.7% between week 3 and month 3 after KTx; and 18.20 ± 11.48% between 3 and 6 months after transplantation.

In comparison, mean lymphocyte counts in the basiliximab group were 23.77 ± 10.42% before Tx and 13.92 ± 8.20% between day 7 and week 3, both significantly higher than in the alemtuzumab group, *p* < 0.0001.

Higher recipient age showed a significant correlation with a lower relative lymphocyte count 3 months after KTx (*p* = 0.032) in the alemtuzumab group. More than sixty percent of all patients in both groups were positive for CMV-IgG, 62.67% versus 70.73% in the basiliximab group (*p* = 0.135). In the alemtuzumab group, the DGF rate in CMV positive patients was significantly higher when compared to CMV negative recipients (28.12% versus 46.85%, *p* = 0.0014). CMV status of the recipients correlated significantly with the pre-Tx lymphocyte count, *p* = 0.009. Patients positive for CMV-IgG had a significantly higher lymphocyte count prior to administration of alemtuzumab, when compared with patients negative for CMV-IgG, 24.71 ± 1.01% versus 21.31 ± 3.48%, *p* = 0.029.

Further, women in the alemtuzumab group, who were positive for CMV-IgG, had a significantly higher lymphocyte count prior to KTx (24.07 ± 0.86% versus 20.79 ± 1.05%, *p* = 0.016) and 3 weeks after transplantation (2.49 ± 0.82% versus 2.41 ± 0.21%, *p* = 0.023) when compared to female recipients negative for CMV-IgG. There was no difference for these factors in the basiliximab group.

HLA match/mismatch and PRAs as well as pre- and postoperative serum creatinine levels had no impact on short-term outcome or relative lymphocyte counts.

Recipients who developed DGF after induction therapy with alemtuzumab had a higher lymphocyte count within the first 3 weeks after Tx than the patients without DGF, 3.03% ± 3.78 versus 2.45% ± 2.82. These early post-Tx lymphocyte counts equate to 13.13% (DGF group) and 10.7% (non-DGF group) of the pre-Tx counts, which showed a significant difference *p* = 0.036. Despite the high rate of DGF in the basiliximab group, no such correlation could be found there. In the control group, the lymphocyte counts within the first 3 weeks after KTx were 12.29 ± 1.35% in the DGF group and 15.10 ± 1.26% in the non-DGF group, *p* = 0.148.

### 3.2. Acute Rejection

Acute rejection defined as either biopsy proven (BPAR) or clinically suspected occurred with a significantly higher incidence in the basiliximab group (23 (11.06%) versus 12 (5.33%), *p* = 0.0372). In the alemtuzumab group, percutaneous kidney biopsies revealed 3 Banff II rejections in the DGF subgroup and 2 Banff II rejections in the non-DGF subgroup. In the control group, 10 acute rejections of the allografts were clinically suspected and 13 had a BPAR (4 Banff I and 9 Banff II rejections). Banff score was determined according to the Banff 07 classification of renal allograft pathology, published in the American Journal of Transplantation 2008 [19]. Fourteen rejections occurred in the DGF group and 9 in the non-DGF group. All of them could be treated successfully with steroids and an increase of maintenance immunosuppressive therapy. Acute rejection did not show any correlation with CMV status.

### 3.3. Patient and Graft Survival

**Figure 1:**
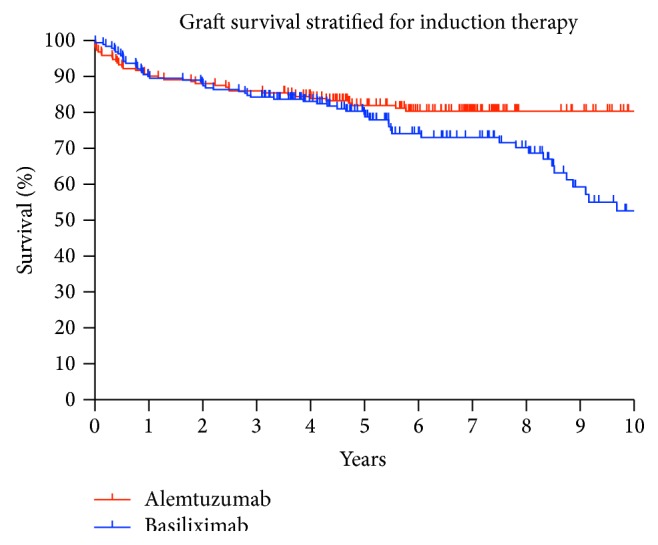
Graft survival after 1, 5, and 10 years was 90.16%, 81.94%, and 80.36% in the alemtuzumab group versus 89.52%, 78.52%, and 52.66% for KTx recipients who received basiliximab; *p* = 0.076.

**Figure 2:**
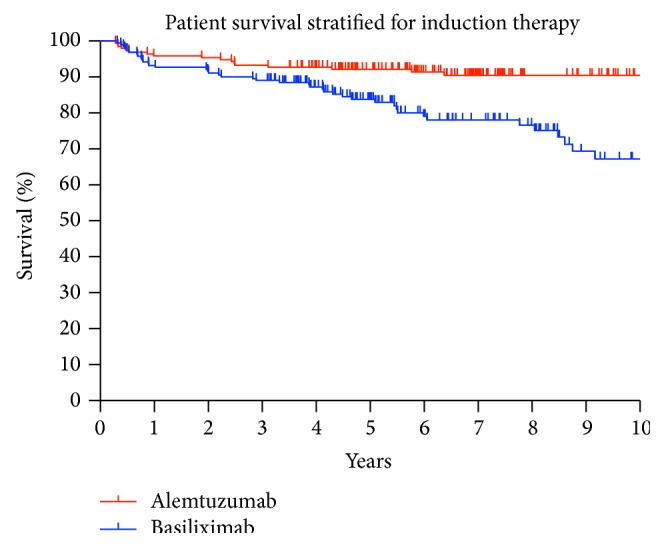
Patient survival after 1, 5, and 10 years was 95.85%, 92.16%, and 90.43% in the alemtuzumab group versus 92.67%, 83.77%, and 62.25% for KTx recipients who received basiliximab; *p* = 0.001.

Five- and ten-year graft survival differed between the alemtuzumab (81.94% and 80.36%) and basiliximab group (78.52% and 52.66%) but without reaching significance, *p* = 0.076, Figure 1. The patient survival in the alemtuzumab group was significantly better five and ten years after KTx compared to the basiliximab group, 92.16% and 90.43% versus 83.77% and 62.25%, *p* = 0.001, Figure 2.

Furthermore, a lymphocyte count below 2.5% during the first three weeks after KTx resulted in a lower graft and patient survival, independently from the induction agent. Five-year graft survival was 79.74% versus 86.21% in the recipient group with a lymphocyte count above 2.5%, *p* = 0.262. The five-year patient survival reached also a higher percentage in the recipient group with more than 2.5% lymphocytes, 91.01% versus 94.83%, *p* = 0.239.

### 3.4. Discussion

Alemtuzumab is a powerful pan-lymphocyte-depleting induction agent [12]. The regeneration of blood lymphocytes and their subpopulations after treatment with alemtuzumab has been described in patients treated for rheumatoid arthritis [16, 20]. In kidney transplantation, alemtuzumab has been primarily used with the aim of reducing maintenance immunosuppression and reducing acute rejection and delayed graft function [2, 11, 21, 22]. Currently, approximately 80% of all kidney transplant patients in the United States receive an antibody induction therapy at the time of transplantation [1, 23, 24]. Lymphocyte depletion for induction has three conceptual possible benefits: inhibition of ischemia reperfusion injury, reduction of consecutive maintenance immunosuppression, and an overall more tolerogenic lymphocyte phenotype after recovery (this remains hypothetical). Several clinical studies have demonstrated that transient T-cell depletion can be combined with steroid-free maintenance immunosuppression with good results. Some centers, however, have recently reported an increasing rate of chronic allograft nephropathy subsequent to aggressive immunosuppression reduction or withdrawal [25].

In an effort to better understand the clinical impact of lymphocyte-depleting induction therapy, the aim of our retrospective association study was to evaluate patient demographics and outcome in light of postoperative lymphocyte depletion and recovery.

CMV represents one of the major pathogens associated with patient survival and long-term graft function [26]. Although CMV status and recipient age are established risk factors in solid organ transplantation, we herein provide evidence that both CMV status and age have an impact on lymphocyte count in kidney transplantation. Macedo et al. investigated the long-term effect of alemtuzumab on T-memory and regulatory subsets after KTx. Findings from this trial indicate an association between effector memory T-cell predominance and increased alloimmune response late after lymphodepletion in KTx [27].

Apart from the retrospective nature of our study hampered by all limitations of this type of analysis, in this study, we have identified a significant relation between DGF occurrence and lymphocyte counts after administering the anti-CD52-antibody alemtuzumab. This correlation could not be detected in the basiliximab group, and hence this effect seems to be correlated with the type of induction treatment. At this point, it should be emphasized that deviations in the demographics of the two groups may have had an impact on the results and limit the conclusions drawn from this trial. Nevertheless, the induction treatment seems to have a major impact on DGF and CMV. Cytomegalovirus represents one of the most important single pathogens in solid organ transplantation and CMV-related complications in organ and composite tissue transplantation have been well documented. However, CMV has not been analyzed in the context of lymphocyte recovery and the occurrence of DGF after depleting therapy for KTx. Furthermore, our data revealed a relationship between age, female gender, and lymphocyte counts after induction therapy with alemtuzumab.

Trzonkowski et al. investigated the homeostatic repopulation by CD28-CD8+ T-cells in alemtuzumab-depleted kidney transplantation recipients. The study demonstrated that CD28-CD8+ T-cells increase in proportion over CD4+ T-cells. This may contribute to a status of compromised immunity, which allows the minimization of maintenance immunosuppressive therapy after alemtuzumab induction [28]. Immunosuppressive protocols with early introduction of an mTor-inhibitor in a calcineurin inhibitor sparing protocol after alemtuzumab induction resulted in an increase in T-reg cells [29]. Hester et al. assessed T-regulatory cells and Th1/Th17 responses in 10 kidney recipients, more than 4 years after alemtuzumab. Their data indicate that a history of rejection and long-term immunosuppressive therapy have an impact on the number of circulating T-regs and Th17 cells [30]. Results from another trial indicate that prolonged defective thymic output leads to a delayed reconstitution of peripheral CD4+ T-cells after depletion with alemtuzumab in renal transplantation [31]. Several studies have analyzed the phenotypes of T-lymphocytes after alemtuzumab, a small number of studies have assessed B-cells and B-cell recovery, and very few studies have aimed to correlate the clinical course of patients with anti-CD52 treatment and lymphocyte recovery.

Cherukuri et al. analyzed the peripheral B- and T-lymphocyte phenotypes of patients after alemtuzumab induction for kidney transplantation. The relationship between peripheral lymphocyte phenotype and graft function was examined and lower numbers of B-cells or B subsets following either basiliximab or alemtuzumab induction correlated with inferior graft function [32].

In this trial, CMV status and age were shown to correlate with lymphocyte recovery after alemtuzumab induction therapy. Lymphocyte counts early after transplantation represent a predictive factor for kidney function early after KTx.

## 4. Conclusion

In conclusion, these results indicate that some recipients' characteristics may help to adjust and individualize immunosuppressive therapy in patients treated with alemtuzumab. A prospective analysis of the phenotype and function of lymphocytes after alemtuzumab induction together with a correlation of the clinical course is warranted in order to confirm our findings and define factors impacting on the outcome after alemtuzumab in kidney transplantation.

## Figures and Tables

**Table 1:** Characteristics of 225 (alemtuzumab) and 205 (basiliximab) recipients and solitary kidney transplants.

Characteristic	Alemtuzumab (*n* = 225)	Basiliximab (*n* = 205)	*p* value
Male gender (*n*, %)	140 (62.22%)	136 (66.34%)	0.495
Age in years (mean ± SD)	48.46 ± 12.37	59.57 ± 13.16	<**0.0001**
Cause of renal failure (*n*, %)			
Glomerulonephritis	71 (31.55%)	68 (33.17%)	0.533
Diabetes mellitus	61 (27.12%)	58 (28.29%)	0.512
Polycystic kidney disease	22 (9.78%)	23 (11.22%)	0.856
Others	71 (31.55%)	59 (28.78%)	0.051
prior kidney transplantation (*n*, %)	41 (18.22%)	17 (8.29%)	**0.002**
PRA+ recipients (*n*, %)	143 (63.56%)	131 (63.90%)	**0.021**
PRA at KTx (in %, mean ± SD)	22.93 ± 26.99	15.65 ± 4.25	0.189
HLA-A mismatch	148 (65.78%)	152 (74.14%)	0.199
HLA-B mismatch	180 (80%)	172 (83.90%)	**0.019**
HLA-DR mismatch	169 (75.11%)	166 (80.97%)	0.457
CMV-IgG+ (*n*, %)	141 (62.67%)	145 (70.73%)	0.135
CMV mismatch (R−/D+) (*n*, %)	42 (18.67%)	34 (16.59%)	0.061
Lymphocyte count before Tx (in %, mean ± SD)	22.8 ± 9.41	23.77 ± 10.42	0.412

## Abbreviations

BPAR: Biopsy proven acute rejection
CMV: Cytomegalovirus
DGF: Delayed graft function
KTx: Renal transplantation
PRA: Panel reactive antibody.
